# Mechanisms of human embryo development: from cell fate to tissue shape and back

**DOI:** 10.1242/dev.190629

**Published:** 2020-07-17

**Authors:** Marta N. Shahbazi

**Affiliations:** MRC Laboratory of Molecular Biology, Francis Crick Avenue, Cambridge Biomedical Campus, Cambridge CB2 0QH, UK

**Keywords:** Human embryo, Morphogenesis, Cell fate, Embryonic stem cells, Aneuploidy, Polarisation

## Abstract

Gene regulatory networks and tissue morphogenetic events drive the emergence of shape and function: the pillars of embryo development. Although model systems offer a window into the molecular biology of cell fate and tissue shape, mechanistic studies of our own development have so far been technically and ethically challenging. However, recent technical developments provide the tools to describe, manipulate and mimic human embryos in a dish, thus opening a new avenue to exploring human development. Here, I discuss the evidence that supports a role for the crosstalk between cell fate and tissue shape during early human embryogenesis. This is a critical developmental period, when the body plan is laid out and many pregnancies fail. Dissecting the basic mechanisms that coordinate cell fate and tissue shape will generate an integrated understanding of early embryogenesis and new strategies for therapeutic intervention in early pregnancy loss.

## Abstract (precis)

**Summary:** Dissecting the mechanisms behind cell fate specification, tissue shape changes and their mutual regulation generates an integrated understanding of embryogenesis. Recent technological advances now make this possible in the human embryo.

## Introduction

Developmental biologists are faced with a question of form and function. Starting from a relatively simple spore, seed or egg, multiple cell types arise that perform specific functions. These different cells organise into diverse configurations, leading to the emergence of tissues of a defined form. Development requires mechanical changes in cell and tissue shape, which drive morphogenesis, and gene expression changes, which regulate cell fate decisions and tissue patterning. These two developmental processes are not independent, as changes in cell fate can modify the architecture of tissues, and vice versa (Chan et al., 2017). Mechanochemical feedback at the molecular, cellular and tissue levels coordinates this crosstalk and instructs tissue self-organisation (see Glossary, Box 1) (Hannezo and Heisenberg, 2019).

**Box 1. Glossary**

**Amnion.** An extra-embryonic membrane that protects the developing foetus from mechanical damage and the maternal immune response.

**Amniotes.** A clade of vertebrates in which the embryo develops while being protected by extra-embryonic membranes, including the amnion. It encompasses reptiles, birds and mammals.

**Amniotic cavity.** Fluid-filled sac located between the amnion and the embryo, the function of which is to protect the embryo from mechanical damage and the maternal immune system.

**Blastocoel.** Fluid-filled cavity present in blastocyst stage embryos.

**Blastocyst.** An embryo composed of an outer trophectoderm layer, an inner cell mass and an internal blastocoel cavity.

**Blastomeres**. Cells of the early embryo, formed by cleavage of the zygote.

**Cytotrophoblast.** Trophectoderm stem cell compartment of the human embryo. It plays a key role during blastocyst implantation and it generates differentiated trophectoderm cells such as syncytiotrophoblast and extravillous trophoblast.

**Ectoderm.** Outermost primary germ layer that gives rise to the nervous system, the epidermis and its appendages.

**Embryonic genome activation.** The process whereby zygotic transcription is initiated and takes over the maternally stored mRNAs and proteins. In human embryos, it takes place at the four- to eight-cell stage transition.

**Embryoid body.** Three dimensional aggregates of pluripotent stem cells commonly used to study differentiation.

**Endoderm.** Innermost primary germ layer that gives rise to the epithelial cells of the gastrointestinal, respiratory, endocrine and urinary systems.

**Epiblast.** The pluripotent embryonic tissue that gives rise to germline and soma, as well as the extra-embryonic amnion.

**Epithelial-to-mesenchymal transition.** Cellular program that leads to the loss of epithelial features (i.e. polarised organisation and cell-cell adhesion) and the acquisition of a mesenchymal phenotype. It plays a key role during gastrulation.

**Extra-embryonic ectoderm.** Trophectoderm-derived tissue that forms during early post-implantation in mouse embryos.

**Inner cell mass.** A group of cells located inside the blastocyst that gives rise to the epiblast and the hypoblast.

**Morphogen.** A signalling molecule that determines cell fates in a concentration-dependent manner.

**Mesoderm.** Primary germ layer positioned in between the ectoderm and the endoderm. It gives rise to muscle, bone, connective tissue, blood vessels, red and white blood cells, and the mesenchyme of various organs such as skin, kidney or gonads.

**Primary yolk sac.** Extra-embryonic membrane formed by hypoblast cells around 7 to 8 days post-fertilisation. By E12-15, it gives rise to a secondary yolk sac, the primary functions of which are hematopoiesis and nutrient transport.

**Primitive endoderm (mouse) or hypoblast (human).** An extra-embryonic tissue characteristic of pre-implantation blastocysts that gives rise to the yolk sac.

**Primitive streak.** Transient structure that appears in the posterior epiblast and marks the start of gastrulation.

**Primordial germ cells.** Specialised cells, precursors of the gametes, that are specified in the epiblast of mammalian embryos during early post-implantation development.

**Pro-amniotic cavity.** Luminal cavity that is formed in the mouse epiblast at E5.0. The fusion between the epiblast and extra-embryonic ectoderm cavities leads to the formation of the amniotic cavity by E5.75 in mouse embryos.

**Self-organisation.** Spontaneous generation of ordered structures that results from the interaction between elements that have no previous pattern.

**Sialomucins.** Glycosylated proteins of the mucin family that contain the acidic sugar sialic acid.

**Trophectoderm.** An extra-embryonic tissue present in pre-implantation embryos that gives rise to the placenta.

Molecular motors generate mechanical forces that are transmitted across tissues via the cytoskeleton and cell-cell adhesion proteins (Heisenberg and Bellaiche, 2013). In the embryo, this leads to changes in cell shape and position, to large tissue-scale deformations, and consequently to morphogenesis. Cells sense mechanical cues – a process termed mechanosensation – via extracellular matrix (ECM) adhesion proteins (Schwartz, 2010), cell-cell adhesion proteins (Benham-Pyle et al., 2015), stretch-activated ion channels (Coste et al., 2010), mechanosensitive transcription factors (Dupont et al., 2011) or directly by the nucleus (Kirby and Lammerding, 2018). Once sensed, mechanical cues are transduced into biochemical signals – a process known as mechanotransduction (Chan et al., 2017). The conversion of mechanical cues into biochemical signals leads to changes in gene expression and protein activity that control cell behaviour, cell fate specification and tissue patterning.

Current consensus focuses on two main ideas to explain the emergence of tissue patterns in response to morphogen (see Glossary, Box 1) signals. In the ‘positional information’ model (Wolpert, 1969), the concentration of a morphogen serves as a coordinate of the position of a cell within a tissue. Cells respond to a specific morphogen concentration making a specific fate choice. Therefore, an existing asymmetry is transformed into a pattern of cell fates. On the contrary, in the reaction-diffusion model (Turing, 1952), pattern formation is a self-organising phenomenon. The basic idea behind this model is the presence of a short-range activator that induces its own expression and the expression of a long-range inhibitor. A spontaneous increase in the local concentration of the activator (a symmetry-breaking event) will induce a further increase in the levels of the activator and expression of the inhibitor. However, as the inhibitor has a higher diffusion rate, this will lead to a peak of activation surrounded by a valley of repression. These two mechanisms, positional information and reaction-diffusion, co-exist during development to generate patterns (Green and Sharpe, 2015).

**Fig. 1.**
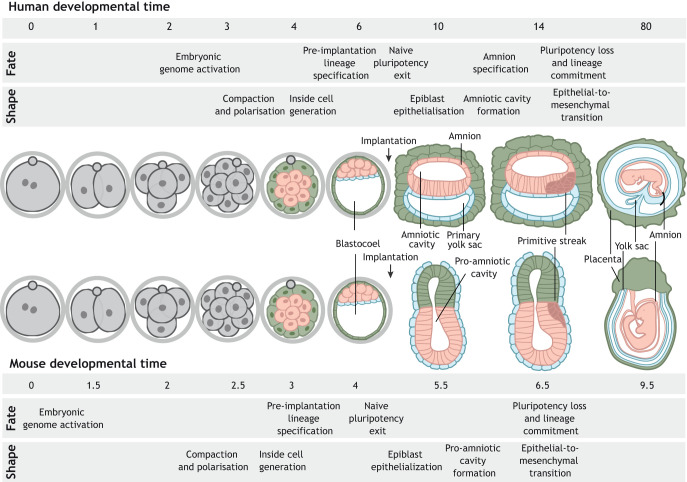
**Overview of human and mouse embryo development.** Upon fertilisation, mouse and human embryos undergo a series of cleavage divisions. The embryonic genome becomes activated by the two-cell stage in mouse embryos and at the four/eight-cell stage transition in human embryos. It is followed by compaction and polarisation, which occur at the eight-cell stage in mouse embryos, and between the eight- to 16-cell stage in human embryos. Formation of a hollow cavity, the blastocoel, denotes the formation of the blastocyst, which, by embryonic day E4 (mouse) and E6 (human), is composed of three main tissues: epiblast, hypoblast and trophectoderm. Upon implantation (E5 in mouse and E7 in human), embryos undergo a global morphological transformation. The embryonic epiblast loses its naïve pluripotent character, becomes epithelial and forms the pro-amniotic cavity (mouse), which spans both the epiblast and the trophectoderm-derived extra-embryonic ectoderm, and the amniotic cavity (human). A key difference between mouse and human embryos relates to the formation of the amnion. Whereas in mouse embryos amnion formation takes place during gastrulation, in human embryos a subset of epiblast cells differentiates to form the squamous amniotic epithelium during early post-implantation (E10). As a result, the human epiblast acquires a disc shape and the mouse epiblast a cylindrical morphology. On embryonic days 6.5 (mouse) and 14 (human), gastrulation is initiated in the posterior epiblast. Cells undergo an epithelial-to-mesenchymal transition, lose their pluripotent character and commit to one of the germ layers. Epiblast-derived tissues are shown in pink, hypoblast-derived tissues are shown in blue and trophectoderm-derived tissues are shown in green.

The use of model organisms allows us to explore the mechanisms that orchestrate the crosstalk between cell fate and tissue shape. For example, cytoskeletal forces lead to the acquisition of cell polarity and the differential segregation of cell fate determinants in *C. elegans* (Goehring et al., 2011), an emerging lumen traps fibroblast growth factor (FGF) molecules, which lead to differentiation in the lateral line primordium of zebrafish (Durdu et al., 2014), and tissue deformations control midgut differentiation in *Drosophila* embryos (Desprat et al., 2008). As human beings, our own development remains the most significant to study, yet is still mysterious due to technical and ethical limitations (Box 2). In this Review, I draw upon knowledge gained from studies in model organisms, embryonic stem cell research and human embryology to propose mechanistic models of three critical developmental events: compaction and polarisation at the cleavage stage; embryonic epithelialisation at the time of implantation; and pluripotent cell differentiation at gastrulation (Fig. 1). The emerging picture supports a role for the crosstalk between tissue shape and cell fate as a determinant of human embryogenesis.

**Box 2. Historical perspective of human embryo development**

The birth of human embryology as a scientific discipline is intimately linked to the creation of human embryo collections (Yamada et al., 2015; Gasser et al., 2014). The pioneering work of Franklin Mall led to the creation of the Carnegie collection in 1887, which harbours more than 10,000 human embryo specimens, and established the basic staging criteria for the developmental classification of human embryos (Keibel and Mall, 1912). Other collections were later created, such as the Kyoto collection, which today holds ∼44,000 specimens (Nishimura et al., 1968). Much of our current textbook knowledge of human development is derived from the early descriptive studies of these samples.

The development of *in vitro* fertilisation (IVF) of human eggs initiated a revolution in human embryo and stem cell research and human reproduction (Edwards et al., 1969; Rock and Menkin, 1944; Shettles, 1955). This initial milestone was followed by the development of conditions to culture fertilised human eggs *in vitro* for up to 5-6 days (Edwards et al., 1970; Steptoe et al., 1971), and ultimately led to the birth of the first IVF baby in 1978, thanks to the tireless efforts of Robert Edwards, Patrick Steptoe and Jean Purdy. Since then, the field of human embryology has flourished. IVF has allowed scientists to describe the dynamics of key morphogenetic processes during early human development, such as cleavage, compaction and blastulation (Wong et al., 2010; Marcos et al., 2015; Iwata et al., 2014); to characterise cell lineage specification events by studying the transcriptional and epigenetic profiles of all the cells present in a developing human embryo (Niakan and Eggan, 2013; Petropoulos et al., 2016; Braude et al., 1988; Zhu et al., 2018); to identify genetic and chromosomal abnormalities that compromise human embryo development (Munne et al., 2009; Vanneste et al., 2009); and, perhaps more importantly, to establish human embryonic stem cell lines (Thomson et al., 1998), which on their own have revolutionised our approach to studying human development and devising regenerative therapies. However, until recently, gene function could not be studied in the context of human embryos. The recent generation of *OCT4* knockout human embryos represents a turning point in the field (Fogarty et al., 2017). This study highlighted differences in gene function between mouse and humans, and established a gold standard for functional studies in human embryos. Thus, human embryology is becoming an experimental science; I argue that, in the years to come, we will witness a surge in the number of mechanistic studies exploring our own development.

## Compaction and polarisation: the first morphogenetic transformation

### Compaction

At the 8- to 16-cell stage, human embryos undergo the process of compaction; blastomeres (see Glossary, Box 1) adhere tightly to each other, maximising their cell-cell contact area, which leads the embryo to acquire a compressed morphology. This process is conserved in all mammalian species studied so far, but the timing varies. The precise timing of the onset and completion of compaction is important for the successful development of both mouse and human embryos (Mizobe et al., 2017; Kim et al., 2017; Skiadas et al., 2006). Human embryos in which compaction is either initiated prematurely or delayed have a lower potential to form blastocysts (see Glossary, Box 1). Premature compaction is associated with cytokinetic failure and multi-nuclear blastomeres (Iwata et al., 2014), whereas delayed compaction is associated with a reduced number of inner cell mass (ICM) cells (see Glossary, Box 1) (Ivec et al., 2011).

The mechanisms that drive compaction in human embryos remain unknown and, instead, have mainly been explored using mouse embryos. The key players are the actomyosin cytoskeleton and the cell adhesion protein E-cadherin. The actomyosin cytoskeleton undergoes pulsed contractions that generate force and increase membrane tension. This happens specifically at the cell-free surface of the embryo, as E-cadherin keeps contractility low at cell-cell adhesion sites (Maitre et al., 2015). An alternative model proposes that E-cadherin-containing filopodia are the major drivers of compaction. By binding to and promoting elongation of the apical surface of neighbouring blastomeres, they trigger the cell shape changes that take place during compaction in a non-cell-autonomous manner (Fierro-Gonzalez et al., 2013). Whether these mechanisms are active in human embryos remains unknown. In terms of timing, studies in mouse embryos suggest that the onset of compaction could be controlled by the decrease in the nuclear/cytoplasmic ratio that takes place during the early cleavage divisions and/or the dilution of a potential inhibitor of compaction present in the zygote (Kojima et al., 2014). These are interesting hypotheses that require further experimental validation.

### Polarisation

Concomitant with the process of compaction, cells acquire apicobasal polarity. In mouse embryos, the basolateral domain concentrates cell adhesion proteins, such as E-cadherin, whereas the apical domain is enriched in apical polarity proteins, such as the partitioning defective (Par) complex and cytoskeletal components (Leung et al., 2016). Recent studies in mice have revealed that protein kinase C (PKC) activation at the eight-cell stage triggers actomyosin polarisation via RhoA (Zhu et al., 2017). In addition, transcription is required for Par complex polarisation. The transcription factors Tfap2c and Tead4 are expressed after embryonic genome activation (EGA, see Glossary, Box 1) and induce the expression of several regulators of the actin cytoskeleton, which become progressively upregulated from the two- to eight-cell stage. This leads to a remodelling of the actin network that is necessary for the clustering of apical polarity proteins, and culminates with the formation of the mature apical domain. As a result, the combined artificial activation of RhoA and expression of Tfap2c and Tead4 is sufficient to induce premature embryo polarisation and to advance morphogenesis (Zhu et al., 2020).

**Fig. 2.**
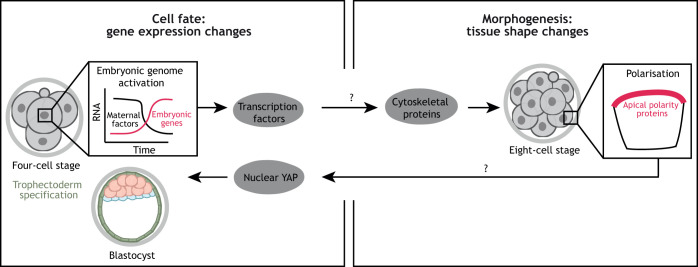
**Proposed cell fate-tissue shape crosstalk during human pre-implantation development.** The onset of apicobasal polarisation at the eight-cell stage may be controlled by the expression of embryonic genes, potentially encoding transcription factors. As recently shown in mouse embryos, embryonic genome activation induces the expression of regulators of the actin cytoskeleton. This leads to a remodelling of the actin network that is necessary for the clustering of apical polarity proteins, and culminates in the formation of the mature apical domain. In turn, the acquisition of apicobasal polarisation may lead to nuclear YAP localisation, as seen in the mouse, and expression of trophectoderm-specific transcription factors. This model remains to be functionally validated in human embryos. Question marks denote molecular connections that have not been validated in human embryos.

Studies in human embryos revealed the presence of apical microvilli and basolateral E-cadherin by the time embryos compact (Alikani, 2005; Nikas et al., 1996), but the exact sequence of events and the mechanisms leading to human embryo polarisation remain to be explored. Given that EGA precedes polarisation, it is tempting to speculate that, as in mouse embryos, EGA controls the timing of human embryo polarisation via as yet unknown transcription factors (Zhu et al., 2020) (Fig. 2).

### The first cell fate decision

Polarisation is fundamental for the divergence of embryonic and extra-embryonic lineages in mouse embryos. In support of this notion, loss-of-function studies demonstrate that Par complex components are needed for the acquisition of an extra-embryonic trophectoderm (TE) (see Glossary, Box 1) fate (Plusa et al., 2005; Alarcon, 2010). Cells that inherit the apical domain differentiate towards TE, whereas cells that do not inherit the apical domain and are internalised become the ICM, and hence the precursors of the embryonic epiblast (see Glossary, Box 1) and extra-embryonic primitive endoderm (PE) (Korotkevich et al., 2017) (see Glossary, Box 1). Inner cell allocation is mainly regulated by apical constriction, heterogeneities in cortical tension and, to a lesser extent, by the orientation of cell division (Samarage et al., 2015; Anani et al., 2014). This leads to the first spatial segregation of cells in the mouse embryo: outer polarised cells and inner apolar cells. Therefore, two morphogenetic cues, polarity and position, regulate the first cell fate decision in mouse embryos.

Mechanistically, the transcription factor Yap is responsible for the ICM-TE bifurcation in mouse embryos (Sasaki, 2015). In polarised cells, Yap translocates to the nucleus and binds the transcription factor Tead4, which leads to the expression of TE genes such as *Cdx2* (Rayon et al., 2014). In turn, Cdx2 inhibits the expression of the ICM-specific pluripotency markers Oct4 (Niwa et al., 2005) and Nanog (Strumpf et al., 2005). By the 32-cell stage, Cdx2+ cells are irreversibly committed to the TE fate (Posfai et al., 2017; Nichols and Gardner, 1984; Gardner, 1983). Yap localisation is regulated by several upstream factors. In apolar cells, the Hippo pathway component angiomotin activates the Hippo kinase Lats, which phosphorylates Yap, leading to its cytoplasmic sequestration. In polarised cells, angiomotin is recruited to the apical domain and becomes inactivated, allowing Yap to translocate to the nucleus (Leung and Zernicka-Goetz, 2013; Hirate et al., 2013). Differences in actomyosin contractility and tension between polar and apolar cells also regulate Yap localisation (Maitre et al., 2016).

Our knowledge of lineage specification in human embryos remains rudimentary. Single-cell sequencing analyses have generated a spatiotemporal catalogue of gene expression and have revealed a number of transcriptional differences between mouse and human embryos (Niakan and Eggan, 2013; Blakeley et al., 2015; Petropoulos et al., 2016; Yan et al., 2013). At the compacted morula stage (embryonic day E4), the transcription of TE genes such as *GATA3* and *PDGFA* is initiated, although cells still express ICM genes (Petropoulos et al., 2016). It is not until the early blastocyst stage (embryonic day E5) when lineage-specific markers start to become mutually exclusive. Aggregation experiments have shown that E5 TE cells still retain the ability to form ICM cells (De Paepe et al., 2013), and, conversely, isolated human ICMs can also generate TE cells (Guo et al., 2020). These results indicate that, even if the ICM-TE lineage segregation takes places at the blastocyst stage (Petropoulos et al., 2016), cells are not yet fully committed.

Analysis of protein expression and localisation has also uncovered interesting differences between mouse and human embryos. YAP localises to the nucleus both in TE and epiblast cells in human blastocysts (Qin et al., 2016), but becomes restricted to the TE at the late blastocyst stage (Noli et al., 2015), in agreement with a delayed ICM-TE segregation in human embryos when compared with mouse. Supporting a potential role for YAP during TE specification, loss of YAP prevents human embryonic stem cell (ESC) differentiation to the TE fate (Mischler et al., 2019; Guo et al., 2020). Surprisingly, *CDX2*, a master TE regulator in mouse embryos, is not expressed until the late blastocyst stage in human embryos (Niakan and Eggan, 2013); its target genes in the mouse, *Eomes* and *Elf5*, are not expressed in pre-implantation human TE (Blakeley et al., 2015); and loss of OCT4 in human embryos leads to a downregulation of CDX2 expression (Fogarty et al., 2017), indicating that, in contrast to mouse embryos, OCT4 and CDX2 do not mutually repress each other in human embryos (Niwa et al., 2005; Ralston et al., 2010). Globally, these findings raise the possibility that different transcription factors control the specification of human TE (Fig. 2). GATA3 is an interesting candidate, as its downregulation in primate embryos inhibits TE differentiation (Krendl et al., 2017) and because it is a downstream target of TEAD4/YAP (Home et al., 2012) that inhibits OCT4 expression (Krendl et al., 2017) in human ESCs differentiated to TE. Future studies are needed to determine whether YAP and GATA3 play a role in TE specification in human embryos. This is especially important as protocols to differentiate human ESCs into TE may not reflect the *in vivo* specification route. Differentiation of human ESCs upon addition of BMP4 has been widely used to obtain TE-like cells (Amita et al., 2013; Xu et al., 2002). However, various reports suggest that cells acquire an extra-embryonic and embryonic mesoderm and/or extra-embryonic amniotic fate rather than a TE fate (Guo et al., 2020; Bernardo et al., 2011). Although new differentiation protocols are being developed (Guo et al., 2020; Mischler et al., 2019; Horii et al., 2019; Dong et al., 2020), these do not reflect the 3D organisation of the embryo, and the potential contribution of embryo polarisation and cell position to TE specification cannot be explored. Therefore, the molecular players involved in human TE specification, and whether, as in the mouse, polarisation controls the first lineage segregation in human embryos remain to be determined.

## The implanting embryo: a global transformation

### The second cell fate decision

Human and mouse embryos reach the blastocyst stage at embryonic days E5 and E3.5, respectively. Blastocyst formation involves a process of cavitation to form the blastocoel (see Glossary, Box 1). In mouse embryos, blastocoel formation is driven by the accumulation of pressurised fluid between cells. This fluid moves from the outside medium into the intercellular space as a consequence of an osmotic gradient, leading to the formation of microlumens that coarsen to form a single lumen (Dumortier et al., 2019). In turn, luminal pressure leads to increased cortical tension in the TE and tight junction maturation to allow lumen expansion (Chan et al., 2019). This is a perfect example of how mechanical cues, in this case hydrostatic pressure, affect differentiation (Chan and Hiiragi, 2020).

Once the embryo reaches the blastocyst stage, it is ready to implant in the maternal uterus. At this stage, mouse and human embryos undergo a second cell fate specification event: the ICM segregates into pluripotent epiblast and extra-embryonic hypoblast (see Glossary, Box 1). Whereas in mouse embryos the specification of TE cells precedes the segregation of ICM cells into epiblast and PE (the mouse equivalent of the human hypoblast), the timing of epiblast-hypoblast segregation in human embryos is still under debate. Although some studies report a concomitant specification of the three lineages, epiblast, hypoblast and TE, in human embryos (Petropoulos et al., 2016), others have identified an early ICM state that precedes the epiblast-hypoblast split (Stirparo et al., 2018). Upon specification, human hypoblast cells become apicobasally polarised and express apical proteins such as the sialomucin (see Glossary, Box 1) protein podocalyxin (Shahbazi et al., 2017). In mouse embryos, polarisation is required for PE maintenance. Inhibition of atypical protein kinase C (aPKC), a component of the Par complex, or β1-integrin, a cell-ECM adhesion protein, leads to defects in epithelialisation and PE maturation (Saiz et al., 2013; Liu et al., 2009). Studies using human ESCs suggest that aPKC may also control the epiblast-hypoblast lineage segregation. Inhibition of aPKC is needed to maintain naïve pre-implantation-like pluripotent human ESCs (Takashima et al., 2014). These cells are competent to differentiate into extra-embryonic endoderm cells, and this has been proposed to be dependent on aPKC signalling (Linneberg-Agerholm et al., 2019), although this hypothesis remains to be formally validated.

A recent study in mouse embryos revealed an unexpected role for the blastocoel during epiblast-PE specification (Ryan et al., 2019). The authors propose that lumen expansion is needed for correct lineage segregation (Ryan et al., 2019). Interestingly, in isolated ICMs, epiblast and PE progenitors segregate, although with an increased proportion of PE to epiblast cells (Wigger et al., 2017). This result suggests that, although the second cell fate decision can take place in the absence of the blastocoel, it may be required to fine-tune the relative numbers of epiblast and PE progenitors.

### Human embryo culture beyond implantation

Our knowledge of human embryo development at and beyond implantation (E7) is very scarce. It has been mainly limited to the analysis of human embryo specimens from the Carnegie collection (Box 2), which have revealed that implanting blastocysts undergo global morphological remodelling to form a disc-shaped embryo (Hertig et al., 1956). Conventional human embryo culture methods only allow embryo growth and survival up to E6-7 (late blastocyst stage). Co-cultures with endometrial cells have traditionally been used to explore the crosstalk between the embryo and the uterus (Weimar et al., 2013; Lindenberg et al., 1985), but whether the embryo undergoes proper morphogenesis in this setting is unclear. Recently, human embryos have been cultured up to day 13 *in vitro*, thus permitting insight into the major morphological transformations of post-implantation human development – TE differentiation (West et al., 2019), amniotic cavitation (see Glossary, Box 1) and primary yolk sac (see Glossary, Box 1) formation – in the absence of interactions with maternal tissues (Deglincerti et al., 2016; Shahbazi et al., 2016). This culture method has been taken forward by the addition of a 3D matrix, which allows amnion (see Glossary, Box 1) specification and further development up to the gastrula stage (Xiang et al., 2019). These systems open the door to study human embryo development beyond implantation; e.g. they have been used to generate a single-cell transcriptome and methylome map of post-implantation human embryos (Zhou et al., 2019; Xiang et al., 2019). But given the lack of an *in vivo* reference, comparisons with primate embryos are important for validation (Nakamura et al., 2016; Boroviak and Nichols, 2017; Ma et al., 2019; Niu et al., 2019).

### Epiblast epithelialisation and amniotic cavitation

The first transformation that epiblast cells undergo is the formation of an epithelial tissue that lines the amniotic cavity. This process is evolutionarily conserved in all amniotes (see Glossary, Box 1) and is a fundamental prerequisite for gastrulation, lineage specification and developmental progression (Sheng, 2015). Mutations that impair pro-amniotic cavity (see Glossary, Box 1) formation lead to developmental arrest in mouse embryos and failed differentiation in embryoid bodies (see Glossary, Box 1) (Sakai et al., 2003; Liang et al., 2005; Smyth et al., 1999).

In mouse and human embryos, epiblast epithelialisation takes place during implantation (Shahbazi and Zernicka-Goetz, 2018). In response to ECM proteins secreted by the extra-embryonic tissues (Li et al., 2003), epiblast cells become polarised and form a rosette-like structure that undergoes lumenogenesis to form the amniotic cavity (Bedzhov and Zernicka-Goetz, 2014; Luckett, 1975). In the mouse, epiblast polarisation is triggered by β1-integrin signalling and requires phosphatase and tensin homologue (Pten) and integrin-linked kinase (Ilk) activity (Meng et al., 2017; Sakai et al., 2003), whereas lumenogenesis is triggered by exocytosis of apical vesicles and membrane separation (Shahbazi et al., 2017), a process known as hollowing (Bryant and Mostov, 2008). In the human context, further mechanistic insight has been gained through the use of 3D ESC cultures, which recapitulate the processes of epithelialisation and cavity formation (Shahbazi et al., 2016; Taniguchi et al., 2015). Whereas actin polymerisation promotes cavity formation, actomyosin contractility and the activity of Rho-associated protein kinase (ROCK) prevent lumenogenesis (Taniguchi et al., 2015). Accordingly, the addition of a ROCK inhibitor to human and monkey embryo culture promotes amniotic cavity formation (Xiang et al., 2019; Niu et al., 2019). The positive effect of ROCK inhibition during lumenogenesis has been described in other models of hollowing (Bryant et al., 2014), suggesting it is evolutionarily conserved.

Once the incipient amniotic cavity is formed, mouse and human embryos acquire different morphologies (Fig. 1). In the mouse embryo, the TE-derived extra-embryonic ectoderm also undergoes a cavitation event. The subsequent fusion of the epiblast and extra-embryonic ectoderm cavities leads to the formation of the pro-amniotic cavity, which spans both tissues (Christodoulou et al., 2018). In contrast, the human TE gives rise to the cytotrophoblast (see Glossary, Box 1). Epiblast cells in contact with the cytotrophoblast differentiate to form an extra-embryonic tissue, the amnion, whereas epiblast cells opposite the cytotrophoblast remain pluripotent (Shahbazi and Zernicka-Goetz, 2018). As a result, whereas mouse embryos acquire a cylindrical shape, human embryos display a disc-shaped morphology.

### Pluripotency and epiblast shape

Epiblast cells at the blastocyst stage display naïve pluripotency, characterised by global hypomethylation, expression of transcription factors promoting the naïve state and two active X-chromosomes in female cells (Nichols and Smith, 2012; Theunissen et al., 2016). This developmental ‘blank state’ is lost as embryos implant: genes that promote the naïve state are downregulated, whereas post-implantation factors become upregulated, methylation levels increase and one of the X chromosomes is inactivated in female cells (Nakamura et al., 2016; Xiang et al., 2019; Kalkan and Smith, 2014). Importantly, exit from the naïve pluripotent state happens concomitantly with the morphological transformation of the epiblast into an epithelial tissue that lines the amniotic cavity, but whether these two events are mechanistically linked was unknown until recently. Using 3D cultures of mouse and human ESCs, and mouse and human embryo cultures, it has been shown that amniotic cavity formation is transcriptionally controlled by a pluripotent state transition (Shahbazi et al., 2017). Naïve pluripotent cells polarise in response to the underlying ECM, but apical vesicle exocytosis is compromised. Consequently, failure to dismantle the naïve state leads to impaired amniotic cavitation and epiblast epithelialisation both in mouse and human embryos (Shahbazi et al., 2017). In the mouse epiblast, the transcription factors Oct4 and Otx2 induce the expression of podocalyxin, which participates in lumen opening by promoting electrostatic membrane repulsion of apposing membranes during vesicle exocytosis. This transcriptional regulation may not be conserved in human embryos, as OTX2 is weakly expressed in the human epiblast (Xiang et al., 2019), and OCT4 has different functions in mouse and human blastocysts (Fogarty et al., 2017). Therefore, the transcription factors that control amniotic cavity formation in human embryos remain to be determined. Notwithstanding the specific mechanism, this is a very clear example of how a cell identity change is necessary for a change in tissue shape.

**Fig. 3.**
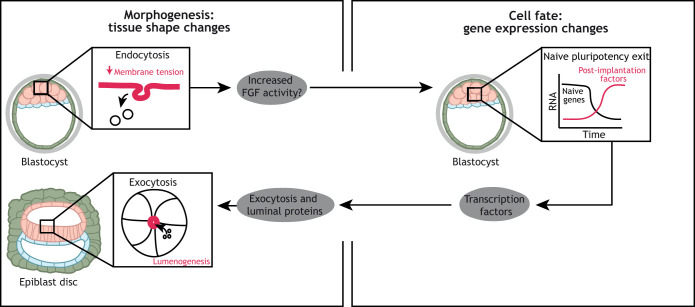
**Proposed cell fate-tissue shape crosstalk during human implantation development.** Upon implantation, epiblast cells exit from the naïve pluripotent state and initiate the expression of post-implantation factors. Studies in mouse ESCs have shown that this is controlled by a decrease in membrane tension, which promotes endocytosis and, as a consequence, increased fibroblast growth factor (FGF) signalling activity, which is required for naïve pluripotency exit. Therefore, membrane tension affects the pluripotent state of a cell. Whether this pathway is active in human embryos remains to be explored. Upon naïve pluripotency exit, genes involved in exocytosis become expressed and initiate the process of lumenogenesis to form the amniotic cavity. Exocytic vesicles provide apical membrane and luminal proteins, which are necessary to build a lumen *de novo*. The transcription factors involved in this process remain to be identified. Question mark denotes a molecular event that has not beem validated in human embryos.

The fate-shape relationship is bidirectional, as changes in cell shape and mechanics also control pluripotent cell identity. Experiments in human ESCs revealed that epithelialisation leads to decreased pluripotent gene expression via integrin β1 activation (Hamidi et al., 2020), and therefore epiblast epithelialisation could facilitate pluripotency exit *in vivo*. In mouse ESCs, a decrease in membrane tension is necessary for naïve pluripotency exit (Bergert et al., 2019), as it allows the activation of FGF signalling, a trigger of naïve exit, via endocytosis of FGF receptors (De Belly et al., 2019). In human ESCs FGF signalling inhibition is implied as a key requirement for naïve human ESCs, and its activation is associated with naïve pluripotency exit (Di Stefano et al., 2018). Thus, it is tempting to speculate that loss of the naïve state in human embryos could also be regulated by changes in membrane tension leading to FGF receptor endocytosis (Fig. 3). Future studies are needed to determine whether similar changes in membrane tension and signalling are observed beyond 2D cultures, using systems that recapitulate both epiblast gene expression and shape. Cell-cell and cell-matrix interactions, as well as the mechanical properties of the ECM, are major regulators of pluripotent stem cell identity *in vitro* (Ranga et al., 2014; Przybyla et al., 2016; Pieters and Van Roy, 2014; Hamidi et al., 2020). Upon naïve pluripotency exit, cells become responsive to mechanical stimuli. In particular, cell stretching leads to an increase in the intracellular levels of calcium, which activate stress and metabolic pathways (Verstreken et al., 2019).

Altogether, these studies provide a glimpse of the complex interplay between transcriptional responses, mechanics, geometry and signalling in pluripotent cells. However, functional studies in the human embryo are needed to determine whether these mechanisms are conserved from ESCs to embryos and from mice to humans.

### Amnion differentiation

A key event in the development of implanting human embryos is the split of epiblast cells into the pluripotent epiblast disc and the differentiated extra-embryonic amniotic epithelium. Our knowledge of the mechanisms that govern this event is very rudimentary, as in most animal models amnion formation takes place at gastrulation (Dobreva et al., 2010). Much of what we know is derived from studies in monkey embryos (Hill, 1932). Upon implantation, epiblast cells in contact with the cytotrophoblast adopt a squamous epithelial morphology and downregulate pluripotency factors such as NANOG and SOX2, whereas epiblast cells in contact with the hypoblast form a columnar epithelium and remain pluripotent (Luckett, 1975; Sasaki et al., 2016). This fate split could be regulated by a gradient of BMP and/or WNT activity, as the monkey cytotrophoblast and amnion are a source of WNT and BMP ligands, whereas the hypoblast secretes WNT and BMP inhibitors (Sasaki et al., 2016). In agreement with this notion, stimulation of human ESCs with BMP leads to the formation of amnion-like cells (Guo et al., 2020; Minn et al., 2020), BMP signalling is required for amnion specification in mouse embryos (Dobreva et al., 2018) and culture of human ESCs in a soft 3D matrix generates amnion-like squamous spheroids in a BMP-dependent manner (Shao et al., 2016). Mechanical cues may cooperate with BMP signalling, as plating human ESCs on a hard surface prevents amniogenesis (Shao et al., 2016). Amnion formation is also regulated by cell density; high cell densities preserve pluripotency and inhibit amniogenesis, whereas low cell densities promote amnion specification (Shao et al., 2017). However, the exact mechanism of amnion specification awaits further investigation.

## Gastrulation: from pluripotency loss to lineage specification

The day 14 rule limits human embryo research to the first 14 days of development (Pera, 2017) and, thus, to the period prior to the formation of the primitive streak (see Glossary, Box 1). Consequently, gastrulation cannot be studied in human embryos. Given that it is ‘not birth, marriage or death, but gastrulation which is truly the most important time in your life’ (Wolpert and Vicente, 2015), it is not surprising that multiple groups are putting a great effort into recreating human embryogenesis using stem cells (Shahbazi et al., 2019; Simunovic and Brivanlou, 2017). During gastrulation, cells undergo concomitant changes in shape and fate. At the site where the primitive streak forms, epiblast cells undergo epithelial-to-mesenchymal transition (EMT) (see Glossary, Box 1), lose their pluripotent features and generate mesoderm (see Glossary, Box 1) and endoderm (see Glossary, Box 1) (Tam and Behringer, 1997). Epiblast progenitors that do not ingress through the streak become ectoderm (see Glossary, Box 1). The onset of gastrulation is regulated by the signalling crosstalk between embryonic and extra-embryonic cells, mediated by the concerted action of BMP, WNT and activin A/Nodal signals and their inhibitors (Tam and Loebel, 2007). These signalling interactions may be further modulated by mechanical cues and the physical structure of the tissue. Modelling the human embryo using stem cells disentangles this complexity by modulating single parameters at a time (Siggia and Warmflash, 2018).

### Self-organisation of human ESCs

In human ESC cultures, cell density and colony shape and size can be precisely controlled using micropattern technology (Théry and Piel, 2009; Bauwens et al., 2008; Nazareth et al., 2013). These initial studies led to the conclusion that colony size and composition affect human ESC identity in the absence of exogenous cues (Peerani et al., 2007).

**Fig. 4.**
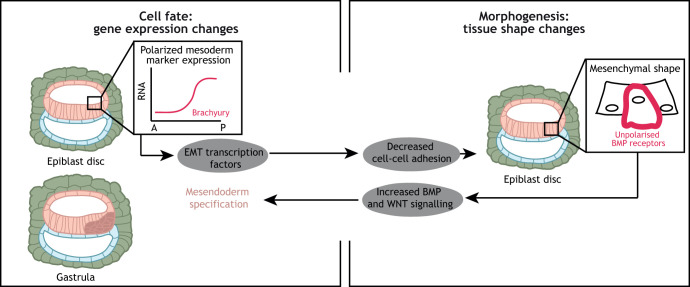
**Proposed cell fate-tissue shape crosstalk during human post-implantation development.** BMP and WNT signals secreted by the amnion and the trophectoderm lead to expression of mesoderm markers, such as the transcription factor brachyury, in the posterior epiblast. Brachyury induces expression of epithelial-to-mesenchymal transition (EMT) transcription factors, which cause a decrease in the levels of E-cadherin. As a consequence, cell-cell adhesions weaken and cells lose their epithelial morphology and their polarised organisation, such as the polarised localisation of BMP receptors. The unpolarised BMP receptors are able to interact with BMP ligands, increasing BMP signalling activity and inducing mesendoderm specification.

Subsequent experiments applied micropattern technology to the study of tissue patterning. When human ESCs are cultured in micropatterns of 0.5 mm, they reach a cell density equivalent to that observed in the pre-gastrulating human epiblast (Etoc et al., 2016). Addition of BMP4 triggers pluripotency exit and lineage commitment. Despite the homogenous presentation of the morphogen, germ layers become radially organised, with TE and amnion on the outside, followed by endoderm, mesoderm and ectoderm towards the centre of the colony (Warmflash et al., 2014; Minn et al., 2020). Mechanistic studies revealed that patterning was a consequence of the diffusion of the BMP4 inhibitor noggin (NOG) and the epithelial architecture of the colony (Etoc et al., 2016). As a result of the epithelial nature of human ESCs, BMP receptors become basally localised and therefore inaccessible to the apically presented BMP4. However, cells in the border of the colony have a less pronounced polarised phenotype, which allows ligand-receptor interactions and, hence, differentiation of the outermost cells. Negative feedback by NOG, which is directly induced by BMP4, is also required to explain the fate patterning (Etoc et al., 2016). Interestingly, a recent report has shown that BMP receptors are basolaterally localised in the early post-implantation mouse epiblast, and this is required for the formation of a robust BMP signalling gradient (Zhang et al., 2019), indicating tissue geometry controls pluripotent cell differentiation both in mice and humans (Fig. 4).

An alternative mechanistic explanation for the emergence of patterning upon BMP4 stimulation has been proposed by Tewary et al. (2017). A reaction-diffusion mechanism is initially responsible for the generation of a self-organised BMP4 gradient. The subsequent interpretation of that gradient and the patterned fate acquisition is consistent with the positional information model. In support of this idea, decreasing the concentration of BMP4 is sufficient to preserve fate patterning in micropatterns of smaller size (0.2-0.3 mm). This represents a potential mechanism to adapt to changes in embryo size, and hence maintain developmental robustness (Tewary et al., 2017). However, a recent report has proposed that BMP4 does not act as a classical morphogen. In very small colonies (one to eight cells), the response to BMP4 is binary: cells adopt either a pluripotent or a TE fate (Nemashkalo et al., 2017). The appearance of mesendodermal fates in a primitive streak-like region additionally requires WNT and Nodal activation (Siggia and Warmflash, 2018).

Self-organised patterns also emerge upon stimulation with WNT (Martyn et al., 2018), which leads to the appearance of an outer mesendoderm ring while inner cells remain pluripotent. Formation of this fate pattern requires E-cadherin cell-cell adhesions, which are more prominent in the colony centre, and dampen WNT activity by sequestering β-catenin. In addition, the WNT inhibitor DKK1 is expressed in response to WNT and controls the size of the mesendoderm ring (Martyn et al., 2019). In agreement with these micropattern studies, a group of cells in the primitive streak of mouse and rabbit embryos expresses both activators and inhibitors of the BMP and WNT signalling pathways (Peng et al., 2016; Idkowiak et al., 2004), indicating there are epiblast-intrinsic mechanisms that regulate the position of the primitive streak.

These examples demonstrate how simplified 2D models of the embryo can help us dissect the chemical control of tissue patterning; but mechanical cues also play a role in patterning. For example, mechanical strains during gastrulation lead to mesoderm specification via induction of the transcription factor brachyury, and this mechanism may be conserved from flies to humans (Brunet et al., 2013). Although the 2D self-organisation of human ESCs described so far does not recapitulate the mechanical features of the developing embryo, recent technological advances provide an opportunity to explore the contribution of mechanics (Vianello and Lutolf, 2019). The use of hydrogels led to the observation that matrix stiffness, degradability and composition influence proliferation, tissue structure and differentiation in the neural lineage (Ranga et al., 2016; Sun et al., 2014). Similarly, mesoderm and endoderm differentiation are enhanced in soft substrates but inhibited in stiffer ones (Przybyla et al., 2016; Chen et al., 2020). Combining micropattern technology with embryo-like compliant hydrogels revealed that gastrulation-like domains emerge in areas of high tension due to the release of β-catenin from cell-cell adhesion sites and its nuclear translocation, both in mouse and human ESCs (Muncie et al., 2020; Blin et al., 2018). To dissect the role of mechanics during development, future experimental strategies need to: be based on the use of robust, reproducible and relevant stem cell models of the embryo (Huch et al., 2017; Van Den Brink et al., 2020); allow mapping of intrinsic stresses and application of extrinsic forces in a precise manner (Vianello and Lutolf, 2019); and use appropriate readouts to assess changes in patterning and morphogenesis.

### Embryonic-extra-embryonic crosstalk

We can add an extra layer of complexity to self-organising models of human ESCs by investigating how extra-embryonic cells modulate human embryonic stem cell fate and shape at gastrulation. This is particularly important given the different organisation of extra-embryonic tissues in mouse and human embryos. Microfluidics has been recently used to model the epiblast-amnion fate split (Zheng et al., 2019). This led to the characterisation of the amnion as a crucial signalling centre. Amniotic epithelial-like cells express high levels of BMP4 and induce gastrulation-like events in a WNT-dependent manner in pluripotent human ESCs and specification of primordial germ cell-like cells (see Glossary, Box 1) (Zheng et al., 2019). Therefore, whereas in mouse embryos the extra-embryonic ectoderm is the key driver of gastrulation (Tam and Behringer, 1997), in human embryos this role may be played by the amnion. Conversely, the function of the hypoblast as an inhibitor of BMP and WNT signalling seems to be conserved in mouse, monkey and humans (Xiang et al., 2019; Stower and Srinivas, 2014; Sasaki et al., 2016). Hypoblast cells secrete BMP and WNT inhibitors in a polarised manner, and therefore the combined action of TE/amnion and hypoblast leads to the generation of a gradient of BMP and WNT activity across the epiblast. Future studies are needed to dissect the specific contributions of the cytotrophoblast, hypoblast and amnion in patterning the human epiblast. This could be achieved by combining studies on *in vitro* cultured human embryos (Xiang et al., 2019) with *in vivo* and *in vitro* cultured monkey embryos (Nakamura et al., 2016; Niu et al., 2019; Ma et al., 2019) and the development of new stem cell models of the human embryo comprising both embryonic and extra-embryonic stem cells, as previously used in the mouse (Harrison et al., 2017; Sozen et al., 2018; Rivron et al., 2018).

## When things go wrong: aneuploidy and pregnancy loss

Understanding the basic mechanisms of human development is fundamental to tackling an important clinical problem: the remarkable inefficiency of human reproduction. It is estimated that only 30-40% of conceptions progress to live birth (Larsen et al., 2013). Development may fail at any time between fertilisation and term, but the early stages are especially crucial. Epidemiological studies estimate that 40-50% of embryos are lost before implantation (Wilcox et al., 2020). Once the embryo implants, the ongoing pregnancy can be detected based on the increased levels of human chorionic gonadotropin (hCG) (Wilcox et al., 1999). Based on hCG serum levels, 20-30% of implanted embryos fail to progress beyond week 6 (Wilcox et al., 1988). Owing to the lack of ultrasound verification, these cases are termed biochemical losses (Larsen et al., 2013; Macklon et al., 2002). The reasons behind this high rate of early pregnancy loss remain poorly understood. Multiple potential mechanisms have been involved, including both alterations in embryo development and maternal causes, but further research is needed to understand their contribution.

The observation that 50-75% of spontaneous abortions present karyotypic abnormalities and morphological malformations (Philipp et al., 2003; Boué et al., 1975) has resulted in a focus on the genetic causes of pregnancy loss. While the incidence of meiotic aneuploidy in most species is remarkably low, 10-30% of fertilised human eggs are aneuploid due to female meiotic errors (Hassold and Hunt, 2001; Nagaoka et al., 2012). These chromosome segregation errors are more prevalent in young women and in women of advanced age, resulting from whole-chromosome nondisjunction and premature sister chromatid separation, respectively (Gruhn et al., 2019). Why, when and how embryos harbouring specific aneuploidies fail during embryogenesis remains unknown. All single chromosome aneuploidies can potentially form blastocysts, but they do so at a lower rate and with a higher incidence of morphological abnormalities. Aneuploid embryos are more likely to arrest during EGA, to be developmentally delayed and to present a low-quality TE and/or ICM (i.e. few cells and cellular fragmentation indicative of cell death) (Minasi et al., 2016; Fragouli et al., 2013). Moreover, despite forming morphologically normal blastocysts, aneuploid embryos display transcriptional and epigenetic alternations that may compromise their subsequent development (McCallie et al., 2016; Licciardi et al., 2018; Starostik et al., 2020). Autosomal monosomies typically lead to biochemical losses, whereas autosomal trisomies are associated with silent miscarriage (presence of extra-embryonic membranes but lack of embryonic structures) and first-trimester miscarriage (Ljunger et al., 2005; Martinez et al., 2010; Ferro et al., 2003; Stephenson et al., 2002). In addition, alterations in the number of chromosomes can be a consequence of mitotic alterations during the first three cleavage divisions, which lead to the formation of mosaic human embryos (Popovic et al., 2019; Starostik et al., 2020; Mantikou et al., 2012). Given the inaccessibility of human embryos at these stages, several fundamental questions remain to be addressed. How do specific alterations in the number of chromosomes impact on morphogenesis or cell fate specification? Is the response to aneuploidy different in embryonic and extra-embryonic cells? What is the fate of aneuploid cells in mosaic embryos? Healthy babies can potentially be born from mosaic aneuploid human embryos (Greco et al., 2015), suggesting aneuploid cells may be selectively eliminated during development. Could this selective elimination be a consequence of the impaired response of aneuploid cells to physical and/or biochemical cues? And given that 25-50% of spontaneous miscarriages do not show chromosomal abnormalities, what is the reason behind their failed development? Could alterations in the cell fate-tissue shape crosstalk be responsible for some of these early pregnancy losses? Addressing these questions and understanding the mechanisms that lead to pregnancy loss is a first step towards the future development of therapeutic interventions.

## Conclusions

Despite remarkable advances in the field of human development, until very recently human embryo research was mainly descriptive and, as Wilhelm Roux already realised by the end of the 19th century, to comprehend the mechanisms of development, we must interfere with it. Today, we are ideally suited to tackle this challenge and decipher how form and function emerge during human embryogenesis. Recent evidence indicates that the crosstalk between cell fate and tissue shape plays an important role during key human developmental events, including compaction and polarisation in pre-implantation embryos, embryonic epithelialisation at the time of implantation and germ lineage commitment at gastrulation. We are just beginning to understand how cell fate specification events modulate tissue organisation and, vice versa, how changes in tissue shape modulate cell identity and tissue patterning, leading to the emergence of self-organisation. A number of crucial questions still remain to be addressed. What are the mechanisms that remodel the implanting human embryo? How do physical and chemical cues coordinate morphogenesis and patterning? What signals trigger cell fate specification during gastrulation? And how are all these events affected when chromosomal alterations are present? To address these questions, we now possess an adequate set of experimental tools: genome editing has already been successfully used to study gene function in human embryos (Fogarty et al., 2017); novel imaging techniques allow us to map cellular behaviours with unprecedented resolution (McDole et al., 2018); new culture methods have extended the window of human development amenable to investigation (Shahbazi et al., 2016; Deglincerti et al., 2016; Xiang et al., 2019); and human ESC-embryo chimeras could be potentially used as a gold standard to study cell potency (De Los Angeles et al., 2015; Alexandrova et al., 2016; Tachibana et al., 2012; Chen et al., 2015), although stringent criteria to assess lineage contribution and ethical considerations need to be applied (Posfai et al., 2020). In addition, human ESCs can mimic different aspects of embryo development *in vitro* (Shahbazi et al., 2019). These stem cell models of the embryo are allowing researchers to deconstruct the complexity of human development and to decipher the basic principles of self-organisation. Last and importantly, the overall increase in assisted reproductive technology cycles over the years, together with the improvement in embryo selection procedures, leads to the generation of vast numbers of surplus human embryos (Kushnir et al., 2017), which represent an invaluable resource for research. Using this new set of tools, we can start to unravel the mysteries of our own development.
